# Tracking Technical Skill Development in Young Basketball Players: The INEX Study

**DOI:** 10.3390/ijerph18084094

**Published:** 2021-04-13

**Authors:** Eduardo Guimarães, Adam D. G. Baxter-Jones, A. Mark Williams, Fernando Tavares, Manuel A. Janeira, José Maia

**Affiliations:** 1 Centre of Research, Education, Innovation and Intervention in Sport (CIFI2D), Faculty of Sport, University of Porto, 4200-450 Porto, Portugal; ftavares@fade.up.pt (F.T.); janeira@fade.up.pt (M.A.J.); jmaia@fade.up.pt (J.M.); 2 College of Kinesiology, University of Saskatchewan, Saskatoon, SK S7N 5B2, Canada; baxter.jones@usask.ca; 3 Department of Health and Kinesiology, College of Health, University of Utah, Salt Lake City, UT 84108, USA; mark.williams@health.utah.edu

**Keywords:** tracking, stability, technical skills, development, youth basketball

## Abstract

This study investigated developmental stability, or tracking, in the development of technical skills in youth male basketball players and retrospectively profiled stable and unstable tracking patterns over time. A total of 97 basketball players were tracked bi-annually over 3 consecutive years. Players were divided into two age-categories according to their age at baseline: under-12; and under-14. Technical skills were assessed using the American Alliance for Health, Physical Education, Recreation and Dance test battery. Anthropometric, body composition, biological maturation and physical performance data were collected. Cohen’s kappa (κ) was used to estimate tracking. With the exception of defensive movement in the under-12 age-category, tracking was low in all skill tests for both under-12 (0.22 ≤ κ ≤ 0.33) and -14 (0.20 ≤ κ ≤ 0.26) groupings. The overall technical skill showed moderate tracking for under-12 players (κ = 0.47) and low tracking for under-14 players (κ = 0.26). At baseline, players who were consistently more skilled or became more skillful (in the under-12 age-category) over time had a better growth-motor performance profile and most of them were selected to be members of regional teams. In conclusion, tracking of individual skill trajectories was low-to-moderate. Moreover, a better growth-motor performance profile seems crucial to maintain high levels of skill performance over time. It is recommended that basketball coaches track the developmental trajectories of their players to better understand the erratic nature of skill development and help design more effective practice regimes.

## 1. Introduction

While coaches are interested in how technical skills develop over time, most previous studies have been cross-sectionally designed and therefore not suitable to describe the development of skill over time [1]. Highlighting the importance of tracking longitudinal development of skill over time. Developmental continuity, or tracking, reflects both the relative stability of inter-individual differences in intra-individual change, as well as the potential to predict future values based on earlier assessments [2,3]. If it is possible to track the development of certain skill indicators, learners who are having difficulties can be identified at an early age and corrective practices introduced at these early ages. In order to develop corrections to skill development, it is important to profile trackers and non-trackers, retrospectively, so that interventions may be successful.

The intermittent, dynamic and intense nature of basketball requires athletes, from an early age, to combine the many and various expressions of their body physique, biological maturation, physiological and psychological attributes as well as tactical skills [4,5,6,7]. However, adequate levels of physical performance and tactical knowledge are apparently not enough to nurture a successful career in basketball. In a sport characterized mainly by the execution of actions such as catching, shooting, passing, dribbling and shuffling, it is crucial that players possess excellent technical skills from an early age [8,9].

The importance of technical skills when identifying and developing athletes with potential has long been recognized by researchers and coaches [10,11,12]. Furthermore, technical skills are apparently more relevant than anthropometrics or physical performance in selection and specialization processes in young basketball players, since they are less dependent on biological maturation differences [13,14,15]. This fact is the most likely reason why the International Basketball Federation, via its affiliated World Association of Basketball Coaches, recommends that youth coaches should be mainly concerned with developing basketball skills [9].

However, despite the importance of technical skill development, most data on young basketball players are derived from cross-sectional study designs. Such cross-sectional studies are unable to consider the intra-individual developmental trajectories as well as inter-individual differences in intra-individual change [1,16]. Longitudinal data are therefore required to adequately describe and interpret unfolding skill trajectories across time. It appears that there is only one published longitudinal study in youth basketball dealing with temporal changes in technical skills. Focusing on macroscopic level changes (i.e., on means) and, therefore, using a team-centered approach, te Wierike et al. [17] reported systematic improvements in ball control across time. However, it is unlikely that all players develop their potential similarly, especially during adolescence where youngsters are known to be more different than alike [18]. Therefore, a distinct developmental picture may emerge when using player-centered approaches to explore microscopic level changes (i.e., inter-individual differences in intra-individual changes). No other published studies are available, either in basketball or other sports, that examine how technical skills unfold across time, or assess the degree to which an individual player tends to keep her/his relative rank position in technical skill development within her/his group over time [2].

The present paper uses the concept of biological developmental canals, first proposed by Waddington [19] and later extended by Tanner [20], to investigate the degree of stability, or tracking, of human growth patterns. Furthermore, human growth is said to be stable, or canalized, if an individual’s serial data remains in a growth canal defined by major centiles. For example, between centiles 5 and 25. In contrast, if the serial growth data of an individual cross two major centile lines then decanalization is said to have occurred [21]. These suggestions can reasonably be extended to players’ technical skill changes if it is considered that skill development can be described in terms of canals. If tracking exists, it is expected that a player’s successive measurements will stay in the same quantile of the distribution of the measurements as that distribution changes over time [2]. Therefore, the sample of skill development values at each time-point can be divided into quartiles or tertiles and a measure of concordance for quartile or tertile membership computed. The Cohen´s κ is a well-suited measure for this purpose [22].

This study will provide novel insights into how youth player’s develop technical skills, particularly as this relates to the identification of basketball players skill stability or canalization (within-participant consistency in upper, middle or lower technical skill canals) and skill positive/negative instability or decanalization (within-participant inconsistency by crossing upwards or downwards canals). Furthermore, it is contended that if it is possible to retrospectively identify the multivariate growth-motor performance profiles of basketball players whose technical skills are highly canalized (i.e., revealing high tracking) or, decanalized, this may be helpful for coaches to design more effective and differential training programs for each distinct developmental group. Although there is a notion that there is considerable heterogeneity in young basketball players’ responsiveness to regular training and competition, most published reports highlight main effects and group differences (team-centered approach) while limited attention has been given to individual differences (player-centered approach) in trainability [23].

This paper has two aims: (i) to track the developmental trajectories of technical skills in under-12 and under-14 male basketball players consecutively over 3 years to identify their consistency (i.e., canalization) or positive and negative developmental instability (i.e., decanalization - when players systematically cross canals); (ii) to retrospectively profile players with (a) a stable tracking pattern, that is, those who consistently remain in upper and lower canals and (b) an unstable tracking pattern, that is, those who consistently showed positive and negative instability between six time-points. Although there are apparently no previously documented footprints available for tracking young athletes, the following hypotheses are proposed: (i) technical skills are expected to track over time (i.e., from moderate-to-high). This prediction is aligned with the expectations of coaches and basketball federations and associations when proposing and planning players’ skill development within age-categories as well as across time; (ii) players from both age-categories who are consistently more skilled over the 3 years (i.e., who are canalized in their technical skill development) will display a best-suited growth-motor performance profile at baseline when compared to less skilled players. That is, they are expected to have accumulated more years in formal basketball training, belonging to a regional team, and show greater body size, advanced biological maturation and be physically fitter. Finally, basketball players from both age-categories who become more skillful over time are expected to already present the aforementioned profile when the study started.

## 2. Materials and Methods

### 2.1. Sample and Design

The data come from the *In Search of Excellence - a Mixed-longitudinal Study in Young Athletes* (INEX) study, carried out in Porto, Portugal from 2017 to 2019 (www.inex-cifi2d.pt). The study design is described in detail elsewhere [15]. In brief, the basketball portion of the INEX study used a 3-year mixed-longitudinal design with five age-cohorts (11, 12, 13, 14, and 15 years) which had 2-year overlaps between age-cohorts, generating 7-years of data on developmental trajectories collected over 3 years.

A total of 293 male basketball players were recruited from a population of 1256 adolescent male players belonging to 20 of the 25 clubs in the Porto Basketball Association. Players were selected at random to participate in the INEX study by their coaches and/or club team managers. Baseline measurements were collected in June 2017 and measurements were repeated bi-annually until December 2019. Assessments were completed during the same time-periods (June and December) within a time window of 15–20 days. Inclusion criteria were that players from cohorts 1, 2, 3 and 4 who had complete data on 6 time-points. A total of 97 male basketball players, from the total sample of 293, fulfilled this criteria and players were divided in two age-categories according to their age at baseline: under-12 (*n* = 50); and under-14 (*n* = 47). The under-12 players from cohorts 1 and 2 were followed consecutively from 11 to 13.5 years and from 12 to 14.5 years, respectively. The under-14 players are from cohorts 3 and 4 and were followed consecutively from 13 to 15.5 years and from 14 to 16.5 years, respectively. Written informed consent was obtained from parents or legal guardians as well as individual assent from each basketball player. The Ethics Committee of the lead institution approved the study (CEFADE 13.2017), and the Porto Basketball Association gave formal permission for data collection.

### 2.2. Technical Skills

Technical skills were assessed using the American Alliance for Health, Physical Education, Recreation and Dance (AAHPERD) test battery [24]. An extensive description of the protocol of each test is presented elsewhere [15]. In brief, the test battery included: (1) speed shot shooting (points)—players shot the ball from five positions, collected their own rebound, dribbled to another designated position and repeated this sequence as quickly as possible over 60 s; (2) passing (points)—players performed chest passes against a wall marked with six targets and retrieved the ball while moving laterally over 30 s; (3) control dribble (s)—players dribbled the ball while running as quickly as possible in an obstacle course defined by six cones; (4) defensive movement (s)—players performed as quickly as possible lateral slides while keeping the basic defensive position and without crossing their feet in a course defined by six cones. Players performed three trials; the first one was a practice trial and the sum of the second and third trials was used as the test result. An overall measure of technical skill was used after transforming individual test results into z-scores and computing an unweighted sum of all z-scores. Signs were reversed in control dribble and defensive movement since in both tests less time represents better performance.

### 2.3. Variables Used in Growth-Motor Performance Profiling

#### 2.3.1. Training Information

Players’ training experience, expressed as years of formal basketball training, was obtained from self-report questionnaires, and the data were validated against registration histories; available from the official website of the Portuguese Basketball Federation (FPB): www.fpb.pt. Players’ membership to the regional teams was obtained from self-report questionnaires and confirmed by official announcements available from the website of the Porto Basketball Association (ABP): www.abp.pt. Players were selected by the ABP’s head and assistant coaches to integrate (i) the under-12 regional team that competed in friendly tournaments, and (ii) the under-14 regional team that competed in the 2017 Portuguese Inter-Associations National Championship.

#### 2.3.2. Anthropometry and Body Composition

Anthropometric measurements were taken according to standard protocols [25]. Height (cm) and sitting height (cm) were measured using a Harpenden stadiometer (Holtain Ltd., Crymych, UK) with a precision of 0.1 cm. Body mass was measured using a bio-impedance scale (Tanita®BC-418MA, Tanita Corp., Tokyo, Japan) with a precision of 100 g; body fat (kg) and fat-free mass (kg) were derived by bio-electrical impedance according to the manufacturer’s formula for adolescent athletes.

#### 2.3.3. Biological Maturation

Biological maturation was assessed by predicting the attainment of peak height velocity (PHV). Years from, or after, attainment of PHV was estimated using a prediction equation from anthropometric measures [26]. The equation uses a specific formula based on age, sex, height, sitting height and body mass to predict years from or after the occurrence of PHV, a variable termed “maturity offset”. A positive (+) maturity offset represents the predicted number of years the participant is beyond their age of attainment of PHV, whereas a negative (–) value represents the predicted number of years before the attainment of their PHV.

#### 2.3.4. Physical Performance

An extensive description of the protocol of each physical performance test is presented in Guimarães et al. [15]. In brief, the following tests were used: (1) handgrip (static strength)—players exerted maximal handgrip strength (kgf) [27]; (2) 5 and 20 m sprint (running speed)—players ran in a straight line at full speed and times at 5 and 20 m were recorded [28]; (3) sit-ups (abdominal muscular strength and endurance)—players performed the maximum number of sit-ups during 60 s [28]; (4) squat jump and countermovement jump (lower body explosive power)—players performed both vertical jumps as advocated by Bosco et al. [29]; (5) 3 kg seated medicine ball throw (upper body explosive power)—players threw the ball straight forward as far as possible (m) while seated sitting on the floor with their legs fully stretched and their backs against a wall [30]; (6) T-test (agility and body control)—players had to run and change directions rapidly in a T-shape pattern [31]; (7) the Yo-Yo IR1 (aerobic capacity)—players performed repeated 40 m (2 × 20 m) runs with a 10 s active recovery period in between [32].

### 2.4. Data Quality Control

Data quality control was ensured following a series of steps: (1) measurements were performed by trained personnel from the lead Faculty; (2) an in-field reliability approach was used such that a random sample of 3–5 players were re-measured every day; (3) reliability estimates were computed. The technical error of measurement was 0.2 cm for height, 0.1 cm for sitting height, 0.1 kg for body mass and 0.3 kg for body fat and fat-free mass. An ANOVA-based one-way random model was used to estimate players’ performance reliability [33], and the intraclass correlations (R) values ranged from 0.91 (speed shot shooting) to 0.98 (defensive movement) for technical skills and from 0.93 (countermovement jump) to 0.99 (3 kg seated medicine ball throw) for physical performance tests; (4) data cleaning was done to control for punching errors in data entry as well as the putative presence of outliers; (5) normality checks in the distributions of all variables were undertaken.

### 2.5. Statistical Analysis

The descriptive statistics (Mean ± SD; Counts and percentages) were calculated in IBM SPSS 26.0 (IBM Corp., Armonk, NY, USA). Tracking was quantified using Cohen’s κ and defined as “if tracking exists, we could expect that an individual’s successive measurement of technical skill will stay in the same quantile of the population distribution as it changes over time” [2] (p. 34). Players’ outcomes at each time-point were divided into tertiles, and a measure of concordance for tertile membership (Cohen’s κ) was computed. Three developmental canals were considered: upper canal, above percentile 66; middle canal, between percentiles 33 and 66; lower canal, below percentile 33. For each skill and overall technical skill, κ-values of under-12 and under-14 basketball players were computed and compared using a chi-square statistic [34]. As advocated by Landis and Koch [35], tracking was classified as follows: κ < 0.40 poor; 0.40 ≤ κ ≤ 0.75 moderate; κ > 0.75 excellent. All analyses were performed in the Longitudinal Data Analysis software [2].

**Figure 1:**
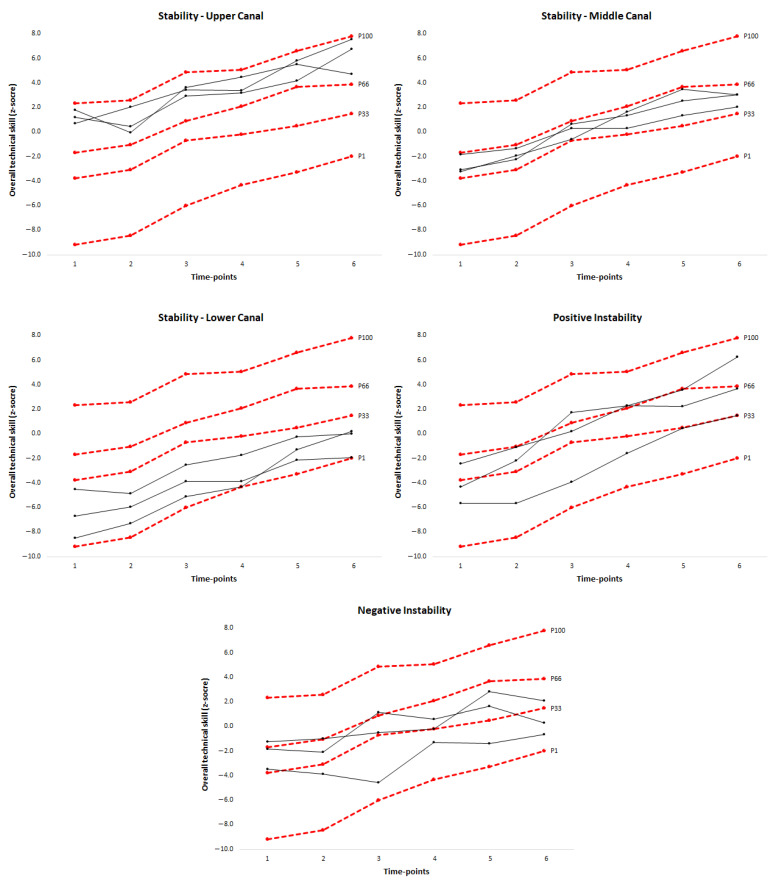
Examples of stability within the upper, middle, and lower canals, and positive and negative instability from 1 to 6 time-point of three young basketball players.

From technical skill canals, the percentage of players’ trajectories with stability (canalization) or instability (decanalization) was computed. Although there were many possible trajectories (729; 3^6^ with three canals and six time-points), our focus was only on players who demonstrated stability within the three canals, as well as positive and negative instability (see Figure 1):

- Stability within the upper, middle or lower canals was identified when a player consistently stayed in each canal from the first to the sixth time-point.
- Positive instability was identified when a player was in the lower or middle canal at the first time-point and moved, respectively, to the middle or upper canal at the sixth time-point or when a player was in the lower canal at the first time-point and moved to the upper canal at the sixth time-point.
- Negative instability was recognized when a player was in the upper or middle canal at the first time-point and moved, respectively, to the middle or lower canal at the sixth time-point or when a player was in the upper canal at the first time-point and moved to the lower canal at the sixth time-point.

Based on overall technical skill development of those players who consistently remained in the upper and lower canals over time, a retrospective profile was created from baseline measurements of training information, anthropometry, body composition, biological maturation and physical performance data. The same profiling approach was performed with those players who showed positive and negative instability over time. Mann-Whitney *U* test and Pearson’s Chi-square test were used to compare for differences between groups in both under-12 and under-14 age-categories. Furthermore, whenever possible, computed Glass rank-biserial correlation [36] and Cramer’s *V* [37] were also computed as measures of effect size. These analyses were performed with IBM SPSS 26.0 (IBM Corp., Armonk, NY, USA).

## 3. Results

The descriptive statistics for technical skills at each time-point for the under-12 and -14 basketball players are shown in Table 1. Overall, and as expected, the basketball players from both age-categories became more skilled over time. There were systematic mean increases in speed shot shooting, passing and overall technical skill, and systematic mean decreases in time to perform the control dibble and defensive movement tests.

Table 2 shows Cohen’s κ tracking values for each technical skill as well as for the overall technical skill for both under-12 and -14 basketball players. Tracking coefficients in all skill tests ranged from κ = 0.22 (poor) in speed shot shooting to κ = 0.40 (moderate) in defensive movement in the under-12 age-category, and from κ = 0.20 (poor) in control dribble to κ = 0.26 (poor) in passing in the under-14 age-category. The overall technical skill showed a moderate κ-value for under-12 players (κ = 0.47) and a poor κ-value for under-14 players (κ = 0.26). Cohen´s κ for under-12 and -14 basketball players did not significantly differ in speed shot shooting (χ^2^ = 0.02; *p* > 0.05) and passing (χ^2^ = 0.22; *p* > 0.05) but were significantly different in control dribble (χ^2^ = 12.70; *p* < 0.001), defensive movement (χ^2^ = 20.66; *p* < 0.001) and overall technical skill (χ^2^ = 31.60; *p* < 0.001).

**Figure 2:**
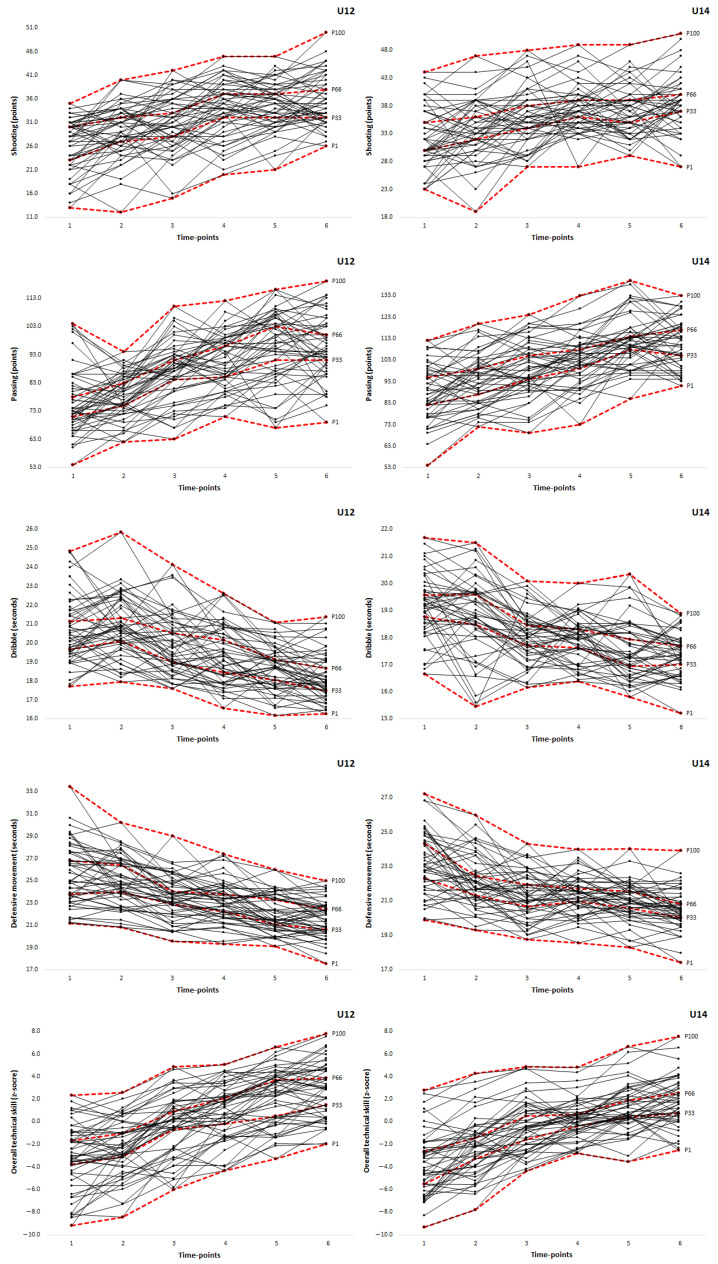
Technical skills developmental trajectories of under-12 and under-14 basketball players.

The percentage of cases showing positive and negative instability (i.e., showing decanalization) was greater than those remaining stable in the upper, middle and lower canals in each skill test, as well as in overall technical skill (see Table 2 and Figure 2). In under-12 players, the percentage of cases within the upper canal ranged from 4.0% (speed shot shooting) to 12.0% (overall technical skill), within the middle canal from 0.0% (speed shot shooting and passing) to 6.0% (overall technical skill), and within the lower canal from 0.0% (speed shot shooting) to 14.0% (passing, defensive movement and overall technical skill). In under-14 players, the percentages within the three canals revealed a similar pattern. In addition, positive instability ranged from 18.0% (overall technical skill) to 28.0% (speed shot shooting) in under-12 players, whereas for under-14 players it varied from 19.1% (overall technical skill) to 29.8% (speed shot shooting). Finally, negative instability varied from 18.0% (overall technical skill) to 26.0% (speed shot shooting) in under-12 players, and from 21.3% (overall technical skill) to 31.9% (control dribble) in under-14 players.

Table 3 and Table 4 show descriptive statistics for growth-motor performance profiling (retrospective training information, anthropometry and body composition, biological maturation and physical performance). As expected, under-12 players in the upper canal (i.e., those who were consistently more skilled) were significantly taller (z = –3.00; *p* < 0.01; r = 1.00), heavier (z = –2.29; *p* < 0.05; r = 0.76) and presented with greater fat-free mass (z = –2.72; *p* < 0.01; r = 0.90) than those players who were less skilled. They were also significantly advanced in their biological maturation (z = –3.00; *p* < 0.01; r = 1.00) and outperformed their peers in all physical performance tests (*p* < 0.05). Additionally, most players in the upper canal – four out of the six – were members of the under-12 regional team (χ^2^ = 6.74; *p* < 0.01; *V* = 0.01). In contrast, no significant differences (*p* > 0.05) were found between players in the upper canal and players in the lower canal in the under-14 age-category. Interestingly, the majority of the players in the upper canal–four out of the five–were members of the under-14 regional team.

The descriptive statistics, at baseline, used for profiling (training information, anthropometry and body composition, biological maturation and physical performance) of under-12 and -14 basketball players with positive and negative instability (i.e., decanalization) in overall technical skill are shown in Table 5 and Table 6, respectively. In the under-12 age-category, significant differences were only found in training experience, favoring the basketball players with negative instability (z = –3.15; *p* < 0.01; r = 0.86), and in fat-free mass favoring the players with positive instability (z = –1.99; *p* < 0.05; r = 0.56). In contrast, no significant differences (*p* < 0.05) were found between basketball players with positive and negative instability in the under-14 age-category.

## 4. Discussion

In this novel study, the concept of developmental canals, as well as the Cohen’s κ statistic, were used as suitable approaches to track skill developmental trajectories in young male basketball players consecutively over 3 years. In addition, retrospective profiles, at baseline, were compared between players who consistently showed stable (upper *versus* lower canal) and unstable (positive *versus* negative instability) tracking patterns over six time-points. To date, the current authors are not aware of any other published study using the idea of developmental canals, widely applied in human physical growth research, to track athletic development and performance when exploring microscopic level changes (i.e., inter-individual differences in intra-individual changes). Although the approach may better reflect the idea of stability of change patterns as well as predictability [20], this is the first time that such an individual-centered approach has been used in youth athletes. This poses problems when comparing our findings. The discussion is framed around three fundamental questions arising from our hypotheses, to which implications for research and practice are added.

*Do players follow a stable trend (i.e., canalized) in their technical skill development?*

The findings showed a strong instability in individual trajectories over 3 years of skill development. It is difficult to make individual and/or group predictions about players’ skill level at later ages. The statistically significant differences found between κ-values of under-12 and -14 players suggest that younger male basketball players tend to have better skill tracking over time, not only in dribbling and defensive tasks, but also when overall technical skill are considered. Two published reports exist using case study designs that deal with the stability of physical performance across time in adolescent soccer [38] and rugby [39] players. These authors reported varying and highly erratic developmental trajectories in several physical performance components. Moreover, Moran et al. [38] (p. 4) contended that “time around PHV appears to be a key period of development that does not always favour the individual player with both increases and decreases in performance being possible”. This finding may explain why the under-12 basketball players showed slightly more stable patterns; as PHV in the average male is achieved at 14 years of age. It is possible that these players, followed consecutively from 11 to 13.5 years, were not yet affected by rapid growth and maturation processes occurring during adolescence [15,40], which allowed them to be technically more consistent compared to their older peers.

Since the analysis was based on technical skill developmental canals, the novel findings showed a higher number of basketball players with positive and negative instability in each skill test, as well as in overall technical skill, than with stability within the upper, middle or lower canals. Positive instability in those skill tests where the outcome was in points and negative instability in those tests where the outcome was in seconds are highly desirable because it shows that less skilled players at baseline exhibited developmental potential to cross canals and achieve a high skill level 3 years later. Inversely, negative instability in skill tests where the outcome was in points and positive instability in tests where the outcome was in seconds reflected a decrease in technical performance over time. No study has previously reported such data in youth athletes. Similar approaches are only available in research with non-athlete populations. For example, in a sample of adolescents from both sexes aged 10–14 years, Souza et al. [41] reported lower instability percentages in several physical fitness tests as compared to those observed in our technical skill tests (from 1.4% to 7.2% for positive instability and from 0.9% to 6.0% for negative instability). This finding not only confirms that most intra-individual changes in technical skills do not occur in specific canals over time (i.e., strong instability in individual trajectories), but also suggests that skill developmental trajectories are apparently more unstable (i.e., decanalized) when compared to trajectories in physical fitness components.

In turn, results from stability within the three developmental canals revealed a similar pattern for both age-categories, with higher percentage of cases in the best development canals. That is, the upper canal for speed shooting and passing and lower canal for control dribble and defensive movement tests. This finding suggests that, regardless of their age, more skilled young basketball players tend to maintain their relative position more than their technically less developed peers. Coaches should use all this information to target young basketball players exhibiting such distinct developmental patterns and provide them with appropriate skill training regimes and, if needed, supplementary individual training workouts. Furthermore, such knowledge is expected to be helpful for basketball coaches when recruiting and selecting young athletes.

*Are there any systematic differences in the multivariate profiles, at baseline, of players who are consistently stable (i.e., canalization) in the upper and lower developmental canals across time?*

In this study, retrospective data of those basketball players who persistently stayed within the upper and lower canals in overall technical skill were compared. Although care must be taken in interpretation, due to the small sample size of both groups, the findings revealed that those under-12 players who were consistently more skilled over 3 years had better growth-motor performance profiles at baseline, and most of them were members of the under-12 regional team. In contrast, no formal statistical tests could be done between under-14 players who consistently remained in the upper and lower canals. Nonetheless, the mean differences found between the two groups suggested that most skilled under-14 players are more likely to show a better growth-motor performance profile at baseline. In addition, the majority of them were members of the under-14 regional team.

There is evidence, mostly from cross-sectional studies, showing significant positive associations between physical performances and technical skills in young basketball players [42,43]. Also, an optimal combination of body size appears to contribute positively to performances in time-based skills such as dribbling and defensive movement [44]. In contrast, previous reports have shown that biological maturation *per se* is not directly linked to game-related skills [13,14]. However, players selected to be members of youth basketball academies or regionals teams are known for attaining their PHV at an early age compared to their non-selected peers [8,13]. Since maturation plays a key role affecting growth and physical performance [40], it is possible that both features influenced positively not only the skill performance at baseline of those players who consistently remain in the upper canal, but also their ability to maintain high skill levels over time. It is apparently simple to recognize that, for example, great performance in shooting and passing requires high levels of strength and power, whereas good performance in time-related skills as dribbling and defensive movement demands great levels of speed, endurance and agility. Therefore, it is recommended that basketball coaches, as well as strength and conditioning trainers, target players displaying such different skill developmental patterns. They should also invest more time in developing basketball players’ physical capacities, mainly those who remained in the lower canal, since they are linked intrinsically to individual skill developmental trajectories.

*Do players who become skillful over time and those who get worse (i.e., decanalization) have a different multivariate profile at baseline?*

The mean differences between groups revealed two very distinct patterns; at baseline, under-12 basketball players with positive instability had a better growth-motor performance profile, whereas in the under-14 age-category the players with negative stability were those who presented the best growth-motor performance retrospective profile. It was expected that the players who became more skilled over time tended to have greater body size, advanced maturity status and higher levels of physical performance at baseline compared to their peers who got technically worse across time. This pattern of results was identified for all variables in the under-12 age-category. However, contrary to initial expectations, an inverse pattern emerged in the under-14 age-category.

Two interpretations are proposed for the above finding. First, being more athletic does not guarantee high levels of skill performance over 3 years of development. Second, even less physically developed athletes can adequately respond to training and competition, and ultimately achieve higher skill performance levels if the right time and development support is given by their coaches, trainers and clubs. In part, this was what Moran et al. [38] suggested in their case study about developmental trajectories of sprint speed and jumping height in youth soccer. These authors showed that, mainly around PHV, some players who lagged behind their peers rapidly improved and matched or surpassed them, while others saw their superiority completely eradicated. In order to reduce bias during recruitment and selection processes, often resulting in substantial dropouts from basketball at early ages, coaches must be aware that young players display erratic skill developmental trajectories. Then, they should target each player and track individual skill patterns of change in order to plan and design training regimes appropriate to the needs of each player.

This study is not without limitations. First, due to sample specificities, care must be taken when generalizing the findings since the sample is from Porto, Portugal. Although it was predicted that young basketball players from the Porto Basketball Association are relatively similar to those from other regions and countries, it is acknowledged that the sample is not widely representative. Second, the sample size limits the power of the statistical tests. However, similar studies in youth athletes using case study designs reported even smaller sample sizes. For example, both Moran et al. [38] and Cobley et al. [39] only sampled 6 youth soccer and rugby players, respectively. Third, it is recognized that there will always be some shortcomings when assessing basketball-specific skills using the so-called analytic technical skill tests. Although measuring skill performance during games is becoming more common in recent studies, these alternative approaches are not without shortcomings. Fourth, it is acknowledged that obtaining training experience data on players’ years of accumulated formal basketball training might be a limited approach. Yet, by consulting official records, it is likely that more reliable information rather than using written or oral questioning.

## 5. Conclusions

In conclusion, this study revealed instability in individual skill trajectories across adolescence. Moreover, the findings showed that a better growth-motor performance profile was crucial to maintain high levels of skill performance and to become more skillful (in under-12 age-category) over three years of development. In light of such evidence, it is recommended that basketball coaches track the developmental trajectories of their players to understand the erratic nature of skill development in youth. Such an approach would allow them to identify basketball players with distinct skill trajectories across time and, consequently, to design more specific and effective training regimes, as well as to enhance recruitment, selection and development. In the future, researchers should consider using player-centered approaches and give more attention to microscopic levels of change (i.e., inter-individual differences in intra-individual change). Moreover, it is suggested that researchers investigate individual differences in responsiveness to regular training and competition, as well as their putative covariates. The fluctuations in skill performance found in this study may be due to individual differences in trainability.

## Figures and Tables

**Table 1:** Descriptive statistics (Mean ± SD) for each technical skill of under-12 and under-14 basketball players at each time-point.

Technical Skills	Time-Points					
	1	2	3	4	5	6
	June 2017	December 2017	June 2018	December 2018	June 2019	December 2019
*Under-12 (n = 50)*						
Speed shot shooting (points)	26.06 ± 5.57	29.24 ± 5.50	30.84 ± 5.89	33.88 ± 5.89	34.44 ± 4.98	35.66 ± 5.47
Passing (points)	75.94 ± 11.51	78.40 ± 7.82	86.80 ± 10.48	90.68 ± 9.49	95.78 ± 12.52	95.42 ± 11.46
Control dribble (s)	20.67 ± 1.74	20.89 ± 1.60	19.88 ± 1.57	19.38 ± 1.54	18.66 ± 1.20	18.21 ± 1.29
Defensive movement (s)	25.60 ± 2.78	24.96 ± 2.22	23.49 ± 1.94	23.13 ± 1.80	22.09 ± 1.68	21.59 ± 1.65
Overall technical skill (z-score)	–3.18 ± 2.95	–2.36 ± 2.49	–0.31 ± 2.72	0.88 ± 2.42	2.18 ± 2.45	2.79 ± 2.47
*Under-14 (n = 47)*						
Speed shot shooting (points)	32.17 ± 5.60	33.74 ± 5.34	36.00 ± 5.18	37.62 ± 4.12	37.17 ± 4.46	38.32 ± 5.18
Passing (points)	87.66 ± 13.42	93.64 ± 12.09	99.83 ± 13.02	103.26 ± 11.69	111.98 ± 12.15	110.72 ± 11.41
Control dribble (s)	19.18 ± 1.20	18.81 ± 1.44	18.03 ± 0.94	17.93 ± 0.83	17.53 ± 1.09	17.34 ± 0.82
Defensive movement (s)	23.24 ± 1.83	22.15 ± 1.54	21.28 ± 1.37	21.31 ± 1.12	20.98 ± 1.12	20.48 ± 1.19
Overall technical skill (z-score)	–3.41 ± 3.02	–1.77 ± 2.77	0.15 ± 2.49	0.80 ± 1.83	1.82 ± 2.21	2.41 ± 2.33

**Table 2:** The tracking coefficients for each technical skill and overall score for under-12 and under-14 basketball players.

Technical Skills		Cohen’s Kappa		Stability (%)			Instability (%)	
		κ	95% CI	Upper Canal	Middle Canal	Lower Canal	Positive	Negative
Speed shot shooting	u12	0.22	0.19–0.26	4.0	0.0	0.0	28.0	26.0
	u14			8.5	0.0	2.1	29.8	25.5
Passing	u12	0.26	0.23–0.30	8.0	0.0	14.0	22.0	24.0
	u14			6.4	0.0	6.4	21.3	23.4
Control dribble *^,†^	u12	0.33	0.28–0.38	10.0	2.0	6.0	20.0	20.0
	u14	0.20	0.14–0.25	2.1	0.0	6.4	23.4	31.9
Defensive movement *^,†^	u12	0.40	0.35–0.45	8.0	2.0	14.0	20.0	20.0
	u14	0.23	0.18–0.28	2.1	0.0	6.4	21.3	23.4
Overall technical skill *	u12	0.47	0.42–0.52	12.0	6.0	14.0	18.0	18.0
	u14	0.26	0.21–0.31	10.6	0.0	4.3	19.1	21.3

* Cohen´s kappa statistically significant differences between under-12 and under-14 groups (*p* < 0.05); ^†^ = since the outcome was in seconds, the best performances were in the lower canal and skill improvement occurred when instability was negative; CI = confidence interval.

**Table 3:** The descriptive statistics (Mean ± SD; Counts and percentages) for training information, anthropometry, body composition, biological maturation, and physical performance at baseline of under-12 basketball players with stability within upper and lower canals in overall technical skill.

Variables at Baseline of Under-12 Players	Upper Canal (*n* = 6)		Lower Canal (*n* = 7)		Mean Difference	z ^†^	χ^2^	Effect Size ^§^
	Mean ± SD	Count (%)	Mean ± SD	Count (%)				
*Training information*								
Training experience (years)	4.00 ± 0.63		3.29 ± 1.25		0.71	–1.02		0.31
Regional team (yes/no)		4(66.7)/2(33.3)		0(0.0)/7(100.0)	---	---	6.74 **	0.01 ^‡^
*Anthropometry and body composition*								
Height (cm)	162.68 ± 7.51		146.81 ± 5.53		15.87	–3.00 **		1.00
Body mass (kg)	47.30 ± 5.39		39.03 ± 6.24		8.27	–2.29 *		0.76
Body fat (kg)	7.62 ± 1.83		8.66 ± 3.09		–1.04	–0.29		–0.10
Fat-free mass (kg)	39.70 ± 5.66		30.37 ± 3.85		9.33	–2.72 **		0.90
*Biological maturation*								
Maturity offset (years)	–1.13 ± 0.52		–2.28 ± 0.44		1.15	–3.00 **		1.00
*Physical performance*								
Handgrip (kgf)	22.84 ± 2.48		15.15 ± 2.33		7.69	–3.00 **		1.00
5 m sprint (s)	1.18 ± 0.11		1.35 ± 0.08		–0.16	–2.57 *		–0.86
20 m sprint (s)	3.82 ± 0.25		4.19 ± 0.23		–0.37	–2.14 *		–0.71
Sit-ups (repetitions)	38.67 ± 10.69		24.57 ± 3.82		14.10	–2.72 **		0.90
Squat jump (cm)	23.66 ± 4.44		17.36 ± 2.33		6.31	–2.43 *		0.81
Countermovement jump (cm)	25.48 ± 6.24		17.23 ± 2.57		8.26	–2.14 *		0.71
3 kg seated medicine ball throw (m)	3.37 ± 0.46		2.33 ± 0.26		1.04	–3.00 **		1.00
T-test (s)	9.76 ± 0.21		11.40 ± 0.78		–1.64	–3.00 **		–1.00
Yo-Yo IR1 (m)	906.67 ± 74.48		434.29 ± 160.71		472.38	–3.02 **		1.00

* *p* < 0.05; ** *p* < 0.01; ^†^ = z test for the Mann-Whitney statistic; ^§^ = Glass rank-biserial correlation; ^‡^ = Cramer’s *V*.

**Table 4:** The descriptive statistics (Mean ± SD; Counts and percentages) for training information, anthropometry, body composition, biological maturation, and physical performance at baseline of under-14 basketball players with stability within upper and lower canals in overall technical skill.

Variables at Baseline of Under-14 Players	Upper Canal (*n* = 5)		Lower Canal (*n* = 2)		Mean Difference
	Mean ± SD	Count (%)	Mean ± SD	Count (%)	
*Training information*					
Training experience (years)	6.40 ± 2.07		3.50 ± 2.12		2.90
Regional team (yes/no)		4(80.0)/1(20.0)		0(0.0)/2(100.0)	---
*Anthropometry and body composition*					
Height (cm)	175.87 ± 7.23		158.93 ± 9.02		16.94
Body mass (kg)	59.10 ± 8.54		48.50 ± 14.00		10.60
Body fat (kg)	8.82 ± 1.30		8.55 ± 3.46		0.27
Fat-free mass (kg)	50.30 ± 7.46		39.95 ± 10.54		10.35
*Biological maturation*					
Maturity offset (years)	0.73 ± 0.74		–0.52 ± 0.21		1.25
*Physical performance*					
Handgrip (kgf)	31.33 ± 8.69		24.40 ± 9.83		6.93
5 m sprint (s)	1.18 ± 0.09		1.18 ± 0.10		0.00
20 m sprint (s)	3.55 ± 0.18		3.69 ± 0.20		–0.14
Sit-ups (repetitions)	43.40 ± 2.88		37.50 ± 10.61		5.90
Squat jump (cm)	24.67 ± 3.81		25.92 ± 5.49		–1.25
Countermovement jump (cm)	25.52 ± 3.44		26.50 ± 6.31		–0.98
3 kg seated medicine ball throw (m)	4.31 ± 0.75		3.40 ± 0.00		0.91
T-test (s)	9.48 ± 0.78		10.87 ± 0.21		–1.39
Yo-Yo IR1 (m)	1296.00 ± 393.55		660.00 ± 254.56		636.00

**Table 5:** The descriptive statistics (Mean ± SD; Counts and percentages) for training information, anthropometry, body composition, biological maturation, and physical performance at baseline of under-12 basketball players with positive and negative instability in overall technical skill.

Variables at Baseline of Under-12 Players	Positive Instability (*n* = 9)		Negative Instability (*n* = 9)		Mean Difference	z ^†^	χ^2^	Effect Size ^§^
	Mean ± SD	Count (%)	Mean ± SD	Count (%)				
*Training information*								
Training experience (years)	2.33 ± 1.00		4.67 ± 1.12		–2.33	–3.15 **		0.86
Regional team (yes/no)		1(11.1)/8(88.9)		2(22.2)/7(77.8)	---	---	0.40	0.15 ^‡^
*Anthropometry and body composition*								
Height (cm)	158.56 ± 8.06		150.63 ± 8.81		7.92	–1.46		0.41
Body mass (kg)	45.76 ± 7.12		40.72 ± 4.96		5.03	–1.63		0.46
Body fat (kg)	7.48 ± 1.18		7.76 ± 1.67		–0.28	–0.44		0.12
Fat-free mass (kg)	38.30 ± 6.47		32.98 ± 4.36		5.32	–1.99 *		0.56
*Biological maturation*								
Maturity offset (years)	–1.41 ± 0.67		–2.04 ± 0.63		0.63	–1.90		0.53
*Physical performance*								
Handgrip (kgf)	21.97 ± 7.11		18.42 ± 4.29		3.54	–0.97		0.27
5 m sprint (s)	1.23 ± 0.08		1.26 ± 0.10		–0.03	–0.88		0.25
20 m sprint (s)	3.74 ± 0.24		3.87 ± 0.30		–0.12	–1.10		0.31
Sit-ups (repetitions)	29.56 ± 7.54		26.22 ± 5.67		3.33	–0.85		0.23
Squat jump (cm)	23.82 ± 4.13		21.78 ± 4.45		2.04	–1.19		0.33
Countermovement jump (cm)	23.10 ± 4.45		21.49 ± 5.61		1.61	–1.15		0.32
3 kg seated medicine ball throw (m)	3.10 ± 0.49		2.70 ± 0.53		0.41	–1.81		0.51
T-test (s)	10.52 ± 0.74		10.70 ± 0.44		–0.19	–0.22		0.06
Yo-Yo IR1 (m)	786.67 ± 341.17		702.22 ± 294.69		84.44	–0.62		0.17

* *p* < 0.05; ** *p* < 0.01; ^†^ = z test for the Mann-Whitney statistic; ^§^ = Glass rank-biserial correlation; ^‡^ = Cramer’s *V*.

**Table 6:** The descriptive statistics (Mean ± SD; Counts and percentages) for training information, anthropometry, body composition, biological maturation, and physical performance at baseline of under-14 basketball players with positive and negative instability in overall technical skill.

Variables at Baseline of Under-14 Players	Positive Instability (*n* = 9)		Negative Instability (*n* = 10)		Mean Difference	z ^†^	χ^2^	Effect Size ^§^
	Mean ± SD	Count (%)	Mean ± SD	Count (%)				
*Training information*								
Training experience (years)	3.78 ± 2.64		5.90 ± 1.85		–2.12	–1.94		0.52
Regional team (yes/no)		0(0.0)/9(100.0)		2(22.0)/8(80.0)	---	---	2.01	0.33 ^‡^
*Anthropometry and body composition*								
Height (cm)	162.78 ± 8.21		165.39 ± 11.61		–2.60	–0.70		0.19
Body mass (kg)	51.08 ± 6.72		53.30 ± 14.23		–2.22	–0.16		0.04
Body fat (kg)	9.29 ± 2.71		8.92 ± 4.84		0.37	–0.78		0.21
Fat-free mass (kg)	41.80 ± 5.23		44.38 ± 10.52		–2.58	–0.74		0.20
*Biological maturation*								
Maturity offset (years)	–0.48 ± 0.52		–0.06 ± 1.13		–0.42	–0.90		0.24
*Physical performance*								
Handgrip (kgf)	27.59 ± 3.65		28.87 ± 8.58		–1.28	–0.29		0.08
5 m sprint (s)	1.25 ± 0.11		1.20 ± 0.08		0.04	–0.94		0.26
20 m sprint (s)	3.71 ± 0.36		3.61 ± 0.20		0.10	–0.69		0.19
Sit-ups (repetitions)	32.00 ± 10.69		37.70 ± 7.50		–5.70	–1.43		0.39
Squat jump (cm)	22.95 ± 5.92		25.61 ± 3.18		–2.67	–1.47		0.40
Countermovement jump (cm)	23.59 ± 5.53		25.02 ± 4.73		–1.42	–0.82		0.22
3 kg seated medicine ball throw (m)	3.51 ± 0.47		3.82 ± 0.74		–0.30	–1.18		0.32
T-test (s)	10.00 ± 0.46		9.72 ± 0.51		0.28	–1.10		0.30
Yo-Yo IR1 (m)	786.67 ± 413.76		992.00 ± 301.58		–205.33	–1.68		0.46

^†^ = z test for the Mann-Whitney statistic; ^§^ = Glass rank-biserial correlation; ^‡^ = Cramer’s *V*.

## Data Availability

Data are available upon request due to ethical restrictions. Individuals or readers interest in the data should contact Professor José Maia (jmaia@fade.up.pt).
